# Supplementary material for: Explaining socioeconomic inequalities in self-reported health outcomes: The mediating role of perceived life stress, financial self-reliance, psychological capital, and time perspective orientations
Source: PLoS One. 2020 Dec 28;15(12):e0243730. doi: 10.1371/journal.pone.0243730 (PMC7769277; doi:10.1371/journal.pone.0243730)
Supplement: Attachment — Submitted filename: Response to reviewers.docx [file pone.0243730.s002.docx]

**Responses to reviewers**

Reviewer #5: The revised manuscript reads well for most parts. A few minor revisions need to be undertaken.

*We thank reviewer 5 for his time to carefully read the manuscript and the constructive feedback to further improve our manuscript. In the sections below we address the minor issues raised by reviewer 5 point by point.*

1. Abstract. The Result section would benefit from review and rephrasing of key findings. In its current form, it can be misunderstood.

*We agree with the reviewer that the Result section of the Abstract was unclear. We revised this accordingly.*

**Revised section of the Abstract:**

**@@** Results: Highest achieved educational level and being in paid employment showed to play a role in the social stratification within self-reported and self-perceived health outcomes, whereas this was not found for personal monthly disposable income. The association between a lower highest achieved educational level and lower self-perceived health was mediated by lower PsyCap and higher perceived life stress levels. The association between a lower highest achieved educational level and higher levels of self-reported physical health conditions was mediated by less financial self-reliance and higher perceived life stress levels. Although no mediating role was found for time perspective orientations in the association between the measured SEP indicators and health outcomes,negative time perspective orientations were associated with either self-perceived health or self-reported physical health conditions.

2. Introduction. Lines 145-150
a. Need to briefly explain the mediating pathway for lower BMI and Cholestrol levels.

*We realized that the illustration of the results of the study by Luthans et al. was not clearly explained. In fact, this study tested no mediation pathways including BMI and cholesterol levels directly. Neverthless, this study included analyses in two separate steps, where first the association between health-related PsyCap with BMI, cholesterol and health satisfaction was tested. In a separate analyses, this study found that health satisfaction was associated with overall well-being. In the revised version of the introduction we now more clearly explain these results.*

**Revised Introduction section (Lines 148-150).**

@@For example, Luthans et al. [21] showed that increased health-related PsyCap is associated with lower BMI and cholesterol levels, as well as with health satisfaction. In turn, in a separate analysis, health satification showed a significant association with overall well-being in this study.@@

b. Reference 22 is not the most appropriate paper to cite here. Reading through the paper by Rew et al (2016), its Pyscap intervention focus in on young homelessness women with substance abuse issues. These are not the main features of your study participants. Therefore need to replace this reference with another more relevant one, as the statement in your manuscript provides potentially misleading information. Alternatively revise the statement.

*The reference was mentioned just to illustrate that PsyCap can be developed and changing in PsyCap may also lead to changes in health-related behavioral outcomes. We added more information about the study in the Introduction section to avoid to be potentially misleading the reader.*

**Revised Introduction section (Lines 150-158).**

@@ Also, PsyCap has been shown to be open for development. For instance, in a study by Rew, Powell, Brown, Becker & Slesnick [22], the feasibility and efficacy of a brief psychological capital intervention was examined using a quasi-experimental pre-post research design with repeated measures. The brief intervention aimed to reduce health-risk behaviors (alcohol use and sexual risk behavior) in 80 ethnically diverse homeless women by increasing their psychological capital. Study results showed that within this group, substance use decreased significantly over time whereas safe-sex self-efficacy and behaviors significantly increased over time [ 22].@@

3. Typos
Lines: 225-227. Review font.
Line 448: Review for typo.

*The font in lines 225-227 was reviewed and adjusted accordingly as well as the typo in Line 448.*

**Revised line 448 (in the revised version this is line 481)**

In line with hypothesis 3a, the reserves PsyCap and financial self-reliance were positively associated with better self-reported health outcomes (higher self-perceived health and lower self-reported physical health conditions; hypothesis 3a, see Table 1).

4. References
Reference # 35 is incomplete. Need to provide relevant information.

*We provided the complete information for this specific reference in the revised version of our manuscript.*

**Revised reference**

@@35.	Bosma H, Schrijvers C, Mackenbach JP. Socioeconomic inequalities in mortality and importance of perceived control: cohort study. BMJ. 1999;319: 1469–1470. Available: www.bmj.com@@
